# Statistical image processing quantifies the changes in cytoplasmic texture associated with aging in *Caenorhabditis elegans* oocytes

**DOI:** 10.1186/s12859-021-03990-3

**Published:** 2021-02-17

**Authors:** Momoko Imakubo, Jun Takayama, Hatsumi Okada, Shuichi Onami

**Affiliations:** 1 Department of Computational Science, Graduate School of System Informatics, Kobe University, Kobe, Hyogo 657-8501 Japan; 2 Laboratory for Developmental Dynamics, RIKEN Center for Biosystems Dynamics Research, Kobe, Hyogo 650-0047 Japan; 3 Laboratory for Developmental Dynamics, RIKEN Quantitative Biology Center, Kobe, Hyogo 650-0047 Japan

**Keywords:** Oocyte aging, *Caenorhabditis elegans*, Image texture analysis, Statistical image processing, Gray level co-occurrence matrix, Nomarski DIC microscopy

## Abstract

**Background:**

Oocyte quality decreases with aging, thereby increasing errors in fertilization, chromosome segregation, and embryonic cleavage. Oocyte appearance also changes with aging, suggesting a functional relationship between oocyte quality and appearance. However, no methods are available to objectively quantify age-associated changes in oocyte appearance.

**Results:**

We show that statistical image processing of Nomarski differential interference contrast microscopy images can be used to quantify age-associated changes in oocyte appearance in the nematode *Caenorhabditis elegans*. Max–min value (mean difference between the maximum and minimum intensities within each moving window) quantitatively characterized the difference in oocyte cytoplasmic texture between 1- and 3-day-old adults (Day 1 and Day 3 oocytes, respectively). With an appropriate parameter set, the gray level co-occurrence matrix (GLCM)-based texture feature *Correlation* (COR) more sensitively characterized this difference than the Max–min Value. Manipulating the smoothness of and/or adding irregular structures to the cytoplasmic texture of Day 1 oocyte images reproduced the difference in Max–min Value but not in COR between Day 1 and Day 3 oocytes. Increasing the size of granules in synthetic images recapitulated the age-associated changes in COR. Manual measurements validated that the cytoplasmic granules in oocytes become larger with aging.

**Conclusions:**

The Max–min value and COR objectively quantify age-related changes in *C. elegans* oocyte in Nomarski DIC microscopy images. Our methods provide new opportunities for understanding the mechanism underlying oocyte aging.

## Background

Oocyte quality is an important factor in the success of animal development. Oocyte quality decreases with aging, thereby increasing errors in fertilization, chromosome segregation, and embryonic cleavage [1–3]. However, the mechanisms underlying the age-related decrease in oocyte quality remain incompletely understood.

The nematode *Caenorhabditis elegans* is a leading model for studying aging because of its short lifespan (~ 3 weeks) and the conservation of longevity pathways from *C. elegans* to humans [4]. In particular, *C. elegans* has been developed as a model for studying age-related decline in fertility [5]. Mutant analyses using *C. elegans* have revealed various genes and signaling pathways that affect aging [6–8]. The molecular processes involved in the age-related regulation of oocyte quality are shared between *C. elegans* and mammals [9].

**Fig. 1:**
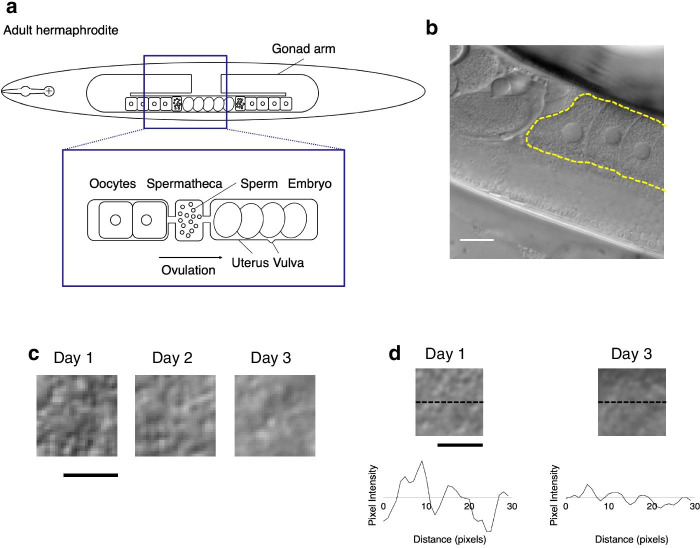
Observation of *C. elegans* oocytes by using Nomarski DIC microscopy. **a** Schematic representation of an adult hermaphroditic gonad. Oocytes mature and enter the spermatheca, where they are fertilized by the accumulated sperm. Embryos are pushed into the uterus and then laid through the vulva. **b** Representative DIC image of oocytes in a gonad. The dotted yellow line surrounds the oocytes. Scale bar 20 μm. **c** Examples of extracted images of the cytoplasmic texture in oocytes from Day 1, Day 2, and Day 3 adults. Scale bar, 5 μm. **d** Cytoplasmic texture images and profile plots of Day 1 and Day 3 oocytes. The dotted black line indicates the position of the horizontal profile plot. Scale bar 5 μm

In *C. elegans* hermaphrodites, sperms are produced during the larval stage and stored in the spermatheca; oocytes are produced continually during the adult stage. Mature oocytes are transported to the spermatheca for fertilization, and the resulting embryos are pushed into the uterus and then laid through the vulva (Fig. 1a). The number of progeny produced on each day decreases with aging and self-reproduction ceases after about 5 days of adult life [10, 11]. In young animals, almost all transported oocytes are fertilized and almost all fertilized eggs are viable; in contrast, older animals produce a substantial number of unfertilized oocytes and inviable eggs, suggesting that oocyte quality declines with aging [9].

Age-related changes in oocytes are found not only in function but also in appearance [2, 5, 9]. In *C. elegans*, aged oocytes shrink, the contacts between oocytes become loose, and oocytes fuse into large clusters [5, 9]. Although there is no method that can objectively quantify age-related changes in oocyte appearance, information provided by such a method could be used to clarify the relationship between changes in the quality of oocytes and their appearance with aging.

Image processing of cell appearance has been applied to various branches of biomedical research, including the identification of malignant cells and detection of cancer [12, 13], analysis of morphological changes [14], and the classification of cell populations with different functions [15, 16]. Texture analysis is one method of classifying biomedical images [17]. In addition, modern machine learning methods, such as deep learning, have recently been applied to various biological applications [18]. The analysis of cell appearance by using image processing is an effective way to characterize and classify the status of cells.

The gray level co-occurrence matrix (GLCM) is a well-known statistical method for examining textures and is widely used to describe spatial properties [19]. The GLCM approach has been used in various biomedical applications, including cell recognition, evaluation of ultrastructural changes, and textural classification of medical images [14, 20–22]. For an image with G gray levels, the GLCM is an estimate of the second-order joint probability *P*(*i, j* | *d, θ*) of two pixels with gray levels *i* and *j* (0 ≤ *i* < G, 0 ≤ *j* < G) that are *d* pixels apart from each other along direction *θ*.

To objectively describe changes in the appearance in oocytes with aging, we used Nomarski differential interference contrast (DIC) microscopy to view and characterize *C. elegans* oocytes. Nomarski DIC microscopes produce contrast by visually displaying the optical phase gradient. DIC microscopy can capture images of transparent objects without chemical staining and is widely used to observe nuclei, nucleoli, and granular structures within *C. elegans* cells [23, 24]. We focused on the cytoplasmic texture because we consider that it reflects the internal status of oocytes more directly than does morphologic appearance, such as the size or shape of oocytes. To quantify the age-associated changes in the cytoplasmic texture of *C. elegans* oocytes, we propose the image feature “Max–min Value” (Mm Value) for measuring textural roughness. We used Mm Value and the GLCM approach to reveal quantitative differences between young and aged oocytes.

## Results

### Mm value reflects age-associated changes in the cytoplasmic texture of *C. elegans* oocytes

To quantify age-associated changes in oocyte appearance, we used Nomarski DIC microscopy to observe the oocytes of 1-, 2-, and 3-day-old *C. elegans* adults (hereafter called Day 1, Day 2, and Day 3 adults, respectively). We observed that the fertilized embryos developed successfully in the Day 1 and Day 2 adults, whereas those in the Day 3 adults exhibited developmental failure, such as errors in egg shell formation or embryonic cleavage (Additional file 1: Movie S1, Additional file 2: Movie S2, and Additional file 3: Movie S3), suggesting that fertility declines in the first 3 days of the reproductive span. We therefore focused on quantifying age-associated changes in oocyte appearance in the DIC images over this 3-day period. In DIC microscopic images, the nucleus appears as a smooth, round region in the center of the oocyte, and the cytoplasm is rough (Fig. 1b). As previously reported [5, 9], we noted various morphologic differences (accumulation of oocytes, oocyte size, cavities, and cluster formation) between Day 1 and Day 3 adults. In addition, visual comparison of the images showed that the oocyte cytoplasmic texture was rougher in appearance in Day 1 than Day 3 worms (Fig. 1c). In a simple comparison of pixel intensities along a line across the oocytes, Day 1 worms showed larger changes in pixel intensity than Day 3 worms (Fig. 1d).

**Fig. 2:**
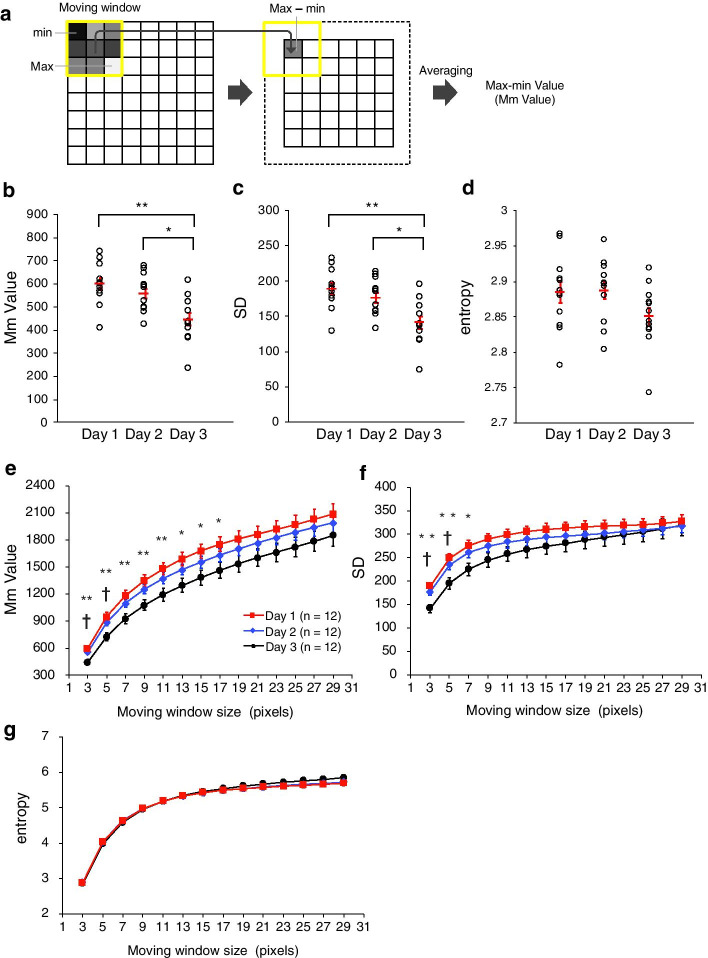
First-order statistical analysis of the age-associated changes in the cytoplasmic texture of *C. elegans* oocytes. **a** Algorithm for calculating the Max–min Value (Mm Value), which is the mean of the difference between the maximum and minimum intensities within each moving window. **b**–**d** Comparison of the first-order statistical features of **b** Mm Value, **c** SD, and **d** entropy (3 × 3-pixel window) between Day 1, Day 2, and Day 3 oocytes. Circles indicate individual animals (n = 12 animals in each age group, pooled from two experiments); red bars indicate the mean values. Error bars indicate SEM. Asterisks indicate statistical significance (*P < 0.05; **P < 0.01; Tukey–Kramer test). **e**–**g** Comparison of **e** Mm Value, **f** SD, and **g** entropy between Day 1, Day 2, and Day 3 oocytes by using window sizes of 3 × 3 to 29 × 29 (n = 12 animals in each age group, pooled from two experiments). Symbols indicate statistical significance (Tukey–Kramer test) between Day 1 and Day 3 oocytes (*P < 0.05; **P < 0.01) or Day 2 and Day 3 oocytes (^†^*P* < 0.05). Error bars indicate SEM

To examine whether the texture changes can be characterized quantitatively, we performed a computational texture analysis of the oocyte cytoplasm. To this end, we defined an image feature, the “Max–min Value (Mm Value),” as follows. Mm Value is calculated by a moving window operation. The maximum and minimum intensities within a moving window of W × W pixels are obtained. Then the difference between maximum and minimum intensities is calculated. Mm Value is defined as the mean of the differences calculated by applying the moving window to the entire image (Fig. 2a). In a rough-texture image, the Mm Value is expected to be high, whereas in a smooth-texture image, the Mm Value is expected to be low.

We observed that the Day 3 Mm Value was significantly smaller than that for Days 1 and 2 when a moving window of 3 × 3 pixels was used (n = 12 animals in each age group; Fig. 2b; and Additional file 4: Fig. S1). Mm Value did not differ significantly between Days 1 and 2. These results suggest that the Mm Value decreases with aging and can be used to quantitatively characterize the age-associated changes in the cytoplasmic texture of *C. elegans* oocytes.

To examine whether general first-order statistics quantitatively characterize the age-associated changes in cytoplasmic texture, we calculated “SD” and “entropy”, which are the mean standard deviation and entropy of local pixel intensities in a moving window. We set the size of the moving window to 3 × 3 pixels. Similar to the Mm Value, the SD was significantly smaller on Day 3 than Days 1 and 2 and did not differ significantly between Days 1 and 2 (n = 12 animals in each age group; Fig. 2c). In contrast, entropy was similar among all 3 age groups (n = 12 animals in each age group; Fig. 2d). Therefore, the change in cytoplasmic texture in aging *C. elegans* oocytes could be characterized quantitatively by using the Mm Value or SD.

To compare the performance of Mm Value, SD, and entropy, we used various sizes of moving window ranging from 3 × 3 to 29 × 29 pixels (Fig. 2e–g). The Mm Value and SD were significantly smaller on Day 3 than Day 1 for smaller window sizes (3 × 3 to 17 × 17 pixels for Mm value and 3 × 3 to 7 × 7 pixels for SD; Fig. 2e, f); the difference between Day 3 and Day 1 was significant for a broader range of window sizes in the case of Mm value. Entropy showed no significant difference between the three age groups at any window size (Fig. 2g).

To examine whether the properties of the first-order statistics are consistent in other datasets, we calculated the Mm Value, SD, and entropy in another dataset (n = 10 animals each at Day 1 and Day 3). As with the first dataset, the Mm Value and SD were significantly smaller on Day 3 than Day 1 for smaller window sizes (3 × 3 to 25 × 25 pixels for Mm value and 3 × 3 to 11 × 11 pixels for SD; Additional file 5: Fig. S2a and b), and the difference between Day 3 and Day 1 was significant for a broader range of window sizes in the case of Mm value. However, unlike with the first dataset, the entropy was significantly smaller on Day 3 than Day 1 when using smaller window sizes (3 × 3 to 11 × 11 pixels; Additional file 5: Fig. S2c).

Our finding that, in both datasets, the Mm Value was significantly smaller on Day 3 than Day 1 for a broader range of window sizes than that observed for SD and entropy (Fig. 2e–g; Additional file 5: Fig. S2) suggests that, compared the other two first-order statistics, Mm Value more robustly characterizes the age-associated changes in cytoplasmic texture.

### The second-order statistic GLCM varies with the age-associated changes in cytoplasmic texture in *C. elegans* oocytes

**Fig. 3:**
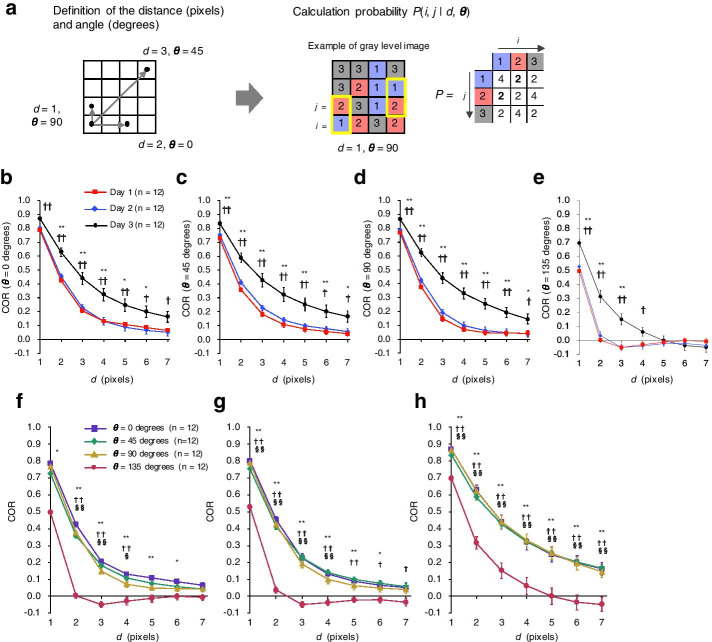
Second-order statistical analysis of the age-associated changes in the cytoplasmic texture of *C. elegans* oocytes. **a** Algorithm for calculating the Gray Level Co-occurrence Matrix (GLCM). First, we define a spatial relationship by using the parameters distance (*d*) and angle (*θ*)*.* We then calculate the second-order joint probability *P*(*i, j* | *d, θ*) of two pixels with gray levels *i* and *j* (0 ≤ *i* < G, 0 ≤ *j* < G). To calculate *P*(*i, j* | *d, θ*), we sum the number of pixels with paired intensities (*i* and *j*) in the defined spatial relationship. For example, when *d* is 1 pixel and the *θ* is 90°, the calculated number of pixels with *i* = 1 and *j* = 2 or *i* = 2 and *j* = 1 is 2. The co-occurrence matrix defined is symmetric. **b**–**e** Effect of oocyte age on *Correlation* (COR). Curves of mean COR as a function of distance *d* for Day 1, Day 2, and Day 3 oocytes are shown for *θ* set at **b** 0, **c** 45, **d** 90, and **e** 135°. Data are means ± SEM (n = 12 animals in each age group, pooled from two experiments). Symbols indicate significant difference (Tukey–Kramer test) in COR between Day 1 and Day 3 oocytes (*P < 0.05; **P < 0.01) or Day 2 and Day 3 oocytes (^†^*P* < 0.05, ^††^*P* < 0.01). **f**–**h** Effect of *θ* on COR. Curves of mean COR as a function of distance *d* when *θ* is set at 0°, 45°, 90°, or 135° are shown for **f** Day 1, **g** Day 2, and **h** Day 3 oocytes. Data are means ± SEM (n = 12 animals in each age group, pooled from two experiments). Symbols indicate significant difference (Tukey–Kramer test) in COR between 0° and 135° (*P < 0.05; **P < 0.01), 45° and 135° (^†^*P* < 0.05; ^††^*P* < 0.01), or 90° and 135° (^§^*P* < 0.05; ^§§^*P* < 0.01)

GLCM is an estimate of the second-order joint probability *P*(*i, j* | *d, θ*) that two pixels with gray levels *i* and *j* are *d* pixels apart from each other in the direction *θ* (Fig. 3a) [19]. To examine whether a second-order statistic more significantly characterizes age-associated texture changes than Mm Value, we used the GLCM-based texture feature *Correlation* (COR), which is a measurement of the gray-level linear dependencies of pixels at specified positions relative to each other. We calculated the COR of the cytoplasmic texture of Day 1, Day 2, and Day 3 oocytes.

As the *d* value increased, COR decreased and converged to around zero (Fig. 3b–e). For various *θ* values, the *d* value at which COR converged to zero was larger in Day 3 oocytes than in Day 1 or 2 oocytes; for example, for *θ* = 135, COR in Day 3 oocytes converged to zero when *d* = 5, whereas COR in Day 1 or Day 2 oocytes converged to zero when *d* = 2 (Fig. 3e). We found that, at several levels of the parameters *d* and *θ*, COR was significantly larger in Day 3 oocytes than in Day 1 and Day 2 oocytes. In particular, the smallest *P* value was obtained for the comparison of COR in Day 3 oocytes versus Day 1 oocytes when *d* = 1 and *θ* = 135 (*P* = 1.0 × 10^–8^; n = 12 animals in each age group; Tukey–Kramer test). The *P* value for this parameter set was four orders of magnitude lower than the lowest *P* value obtained by using Mm Value (window size, 3 × 3 pixels; *P* = 6.0 × 10^–4^; n = 12 animals in each age group; Tukey–Kramer test). These results suggest that COR effectively characterized the age-associated changes in cytoplasmic texture. COR was able to more significantly characterize the differences between Day 1 and Day 3 oocytes than the Mm Value did when using an appropriate parameter set. In addition to COR, we tested several texture-associated features based on GLCM, including *Angular Second Moment* (ASM), *Contrast* (CON), *Inverse Difference Moment* (IDM), and *Entropy* (ENT), but COR continued to yield the best characterization (Additional file 6: Fig. S3).

In general, for all oocytes regardless of age, COR obtained when *θ* = 135 was smaller than that obtained with the other angles tested at equivalent *d* (Fig. 3f–h).

### Sample orientation does not influence the dependency of COR on the angle *θ*

**Fig. 4:**
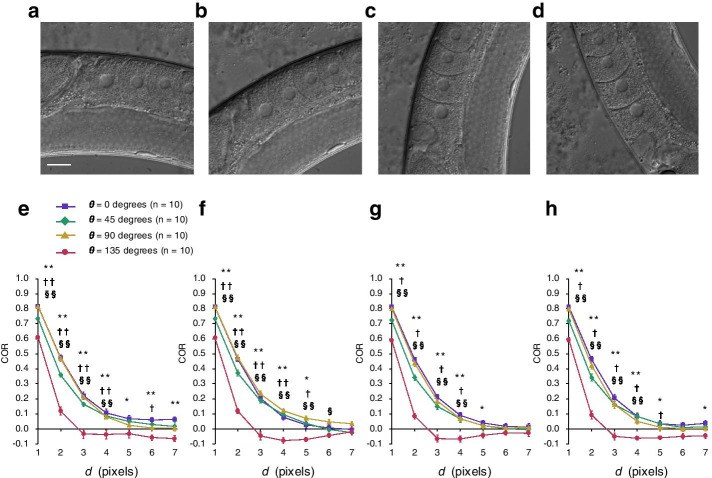
Comparison of COR between angle parameters by using rotated worms. **a**–**d** Representative images of worms rotated **a** 0, **b** 45, **c** 90, or **d** 135° from horizontal (0°). Scale bar, 20 μm. **e**–**h** Effect of worm rotation on COR. Curves of mean COR as a function of distance *d* are shown for worms rotated **e** 0, **f** 45, **g** 90, and **h** 135° from horizontal. Data are means ± SEM (n = 10 animals in each angle group). Symbols indicate significant difference (Tukey–Kramer test) in COR between 0° and 135° (*P < 0.05; **P < 0.01), 45° and 135° (†*P* < 0.05; ^††^*P* < 0.01), or 90° and 135° (^§^*P* < 0.05; ^§§^*P* < 0.01)

Images from DIC microscopy have a shadow-cast appearance oriented in the shear direction of the prism [25]. The texture characterized by using COR was dependent on *θ*, i.e., COR was smaller at *θ* = 135 than at any other angle (Fig. 3f–h). To examine whether this angle dependency is due to either the orientation of the worm or the imaging system itself, we calculated COR after rotating worm samples to 0°, 45°, 90°, or 135° relative to horizontal (i.e., 0°) (Fig. 4a–d). If sample orientation causes the angle dependency, then the angle-dependent property of COR should change depending on sample orientation. Conversely, if the angle dependency is due to the imaging system itself, then orientation should have no effect on this property. We found that, regardless of sample orientation, COR was smaller at *θ* = 135 than at other angles (n = 10 animals in each angle group; Fig. 4e–h). This result indicates that the angle-dependent property of COR measurements of oocyte cytoplasmic texture is due to the imaging system itself, rather than to the orientation of the worm imaged.

### The optimal choice of distance parameter *d* depends on the resolution of the image

**Fig. 5:**
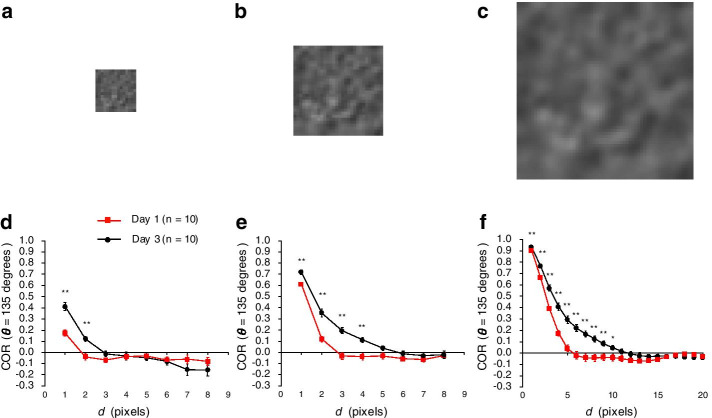
Comparison of COR between pseudo-low and pseudo-high resolution images. **a**–**c** Representative original and rescaled images. The **b** original images (30 × 30 pixels) were **a** downscaled (final size, 15 × 15 pixels) to produce pseudo-low resolution images, and **c** upscaled (final size, 60 × 60 pixels) to produce pseudo-high resolution images. **d**–**f** Curves of mean COR as a function of distance *d* when *θ* = 135 are shown for the **e** original, **d** pseudo-low resolution, and **f** pseudo-high resolution images. Data are means ± SEM (n = 10 animals in each age group; orientation of the worms is 0°)

To examine whether the optimal choice of the GLCM parameter *d* to characterize the age-associated changes depends on the resolution of the image, we calculated the COR in upscaled and downscaled images created by artificially changing the resolution of the original images (Fig. 5a–c). The COR in Day 1 and 3 oocytes converged to zero at smaller *d* in the downscaled images than in the original images (Fig. 5d, e). In contrast, COR converged at larger *d* in the upscaled images than in the original images (Fig. 5e, f). In both the original and rescaled images, the *d* value at which COR converged was larger in Day 3 oocytes than in Day 1 oocytes (Fig. 5d–f). The smallest *P* value was obtained for the comparison of COR in Day 3 oocytes versus Day 1 oocytes in the downscaled, original, and upscaled images when *d* = 1, 3, and 5, respectively (*P* = 8.5 × 10^–5^, 5.7 × 10^–5^, and 5.5 × 10^–5^; n = 10 animals in each age group; Welch’s two-tailed *t* test; Fig. 5d–f). These results suggest that the *d* value at which COR converges to zero and the optimal *d* value to characterize the age-associated changes in cytoplasmic texture increase as the image resolution increases, but the marked difference in the convergence properties of COR between Day 1 and Day3 oocytes is not changed by image resolution.

### Changing smoothness or simulating large structures did not reproduce the age-associated changes in cytoplasmic texture

**Fig. 6:**
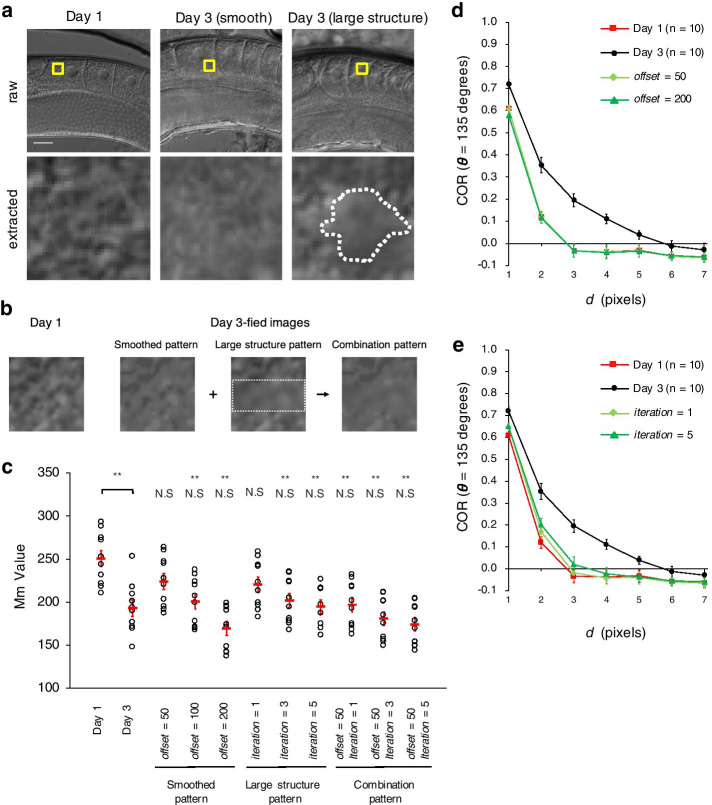
Day 3-fied images created from Day 1 images. **a** Comparison of cytoplasmic texture between Day 1 and Day 3 oocytes. Top, oocyte images; yellow areas indicate representative extracted areas. Scale bar 20 μm. Bottom, images of the extracted areas. The extracted area of the Day 3 oocyte (middle) appears to have a smoother texture than that of the Day 1 oocyte (left). The extracted area of the Day 3 oocyte (right) contains large structures (marked by a dotted white line), whereas that of the Day 1 oocyte is homogeneous. **b** Extracted areas of a Day 1 image and three Day 3-fied images (left, Smoothed pattern; middle, Large Structure pattern; right, Combination pattern) created from the Day 1 image. The dotted white line indicates the filtered area. **c** Mm Values (3 × 3-pixel window) of actual Day 1 and actual Day 3 images, and the three patterns of Day 3-fied images. The Smoothed pattern parameter *offset* was set to 50, 100, and 200. The Large Structure pattern parameter *iteration* was set to 1, 3, and 5. The Combination pattern parameters (*offset*, *iteration*) were set to (50, 1), (50, 3), and (50, 5). Circles indicate the Mm Values of individual animals (n = 10 animals in each age group; orientation of the worms is 0°); red bars indicate the mean values. Error bars indicate SEM. Asterisks indicate statistical significance (Tukey–Kramer test) between actual Day 1 and actual Day 3 images or actual Day 1 and Day 3-fied images (**P < 0.01); N.S., no significant difference between actual Day 3 and Day 3-fied images. **d**, **e** Curves of mean COR as a function of distance *d* when *θ* = 135 are shown for actual Day 1, actual Day 3, and Day 3-fied oocyte images created with **d** Smoothed pattern (*offset* = 50 or 200) or **e** Large Structure pattern (*iteration* = 1 or 5). Data are means ± SEM (n = 10 animals in each age group; orientation of the worms is 0°)

To elucidate the factor that causes the age-associated texture change that is characterized by Mm Value or COR, we visually compared Day 1 and Day 3 oocytes. We considered two factors that might underlie the age-associated texture change characterized by Mm Value and COR: (1) cytoplasmic smoothness in oocytes (i.e., the cytoplasm of Day 3 oocytes appeared smoother than that of Day 1 oocytes) and (2) the distribution of large structures (i.e., large structures were distributed irregularly in the cytoplasm of Day 3 oocytes but the structure was homogenous throughout that of Day 1 oocytes) (Fig. 6a).

To examine whether these factors caused the texture changes, we created ‘Day 3-fied images’ by applying image processing to Day 1 images. We created three patterns of Day 3-fied images by manipulating the factors. The first pattern, ‘Smoothed Pattern,’ was created by smoothing the images of Day 1 oocytes. That is, the maximum and minimum intensities of Smoothed pattern images were normalized to minimum + *offset* and maximum − *offset*, respectively by using the minimum and maximum intensities from Day 1 oocytes. The second pattern, ‘Large Structure Pattern’, was generated by applying a Gaussian filter multiple times to the center part of the Day 1 images; the parameter *iteration* dictates the number of times the filter is applied. We created the third pattern, ‘Combination Pattern’, by first smoothing the Day 1 images and then generating large structures on them (Fig. 6b).

We then compared Mm Value and COR between Day 3-fied, actual Day 1, and actual Day 3 oocyte images (n = 10 animals in each age group; Fig. 6c). We set the parameter *offset* in the Smoothed pattern to 50, 100, or 200 and the parameter *iteration* in the Large Structure pattern to 1, 3, or 5. If the properties of the Day 3-fied images are similar to the actual images, the features of the Day 3-fied images would be expected to be similar to those of actual Day 3 images and differ from those of actual Day 1 images. For Smoothed pattern images, Mm Value at a window size of 3 × 3 pixels for the Day 3-fied images did not differ significantly from actual Day 3 images but was significantly smaller than that of actual Day 1 images (Fig. 6c; *offset* = 100, 200). When the Day 3-fied images were compared with Day 1 images, all of the three patterns of Day 3-fied images reproduced the difference in Mm Value between Day 1 and Day 3 oocytes (Fig. 6c). In addition, for all patterns, the Day 3-fied images reproduced the differences in general first-order statistical features, SD and entropy, between Day 1 and Day 3 oocytes (Additional file 7: Fig. S4). Therefore, in terms of these features, all three patterns of Day 3-fied images were similar to actual Day 3 images.

Next, we calculated COR for the three patterns of Day 3-fied images and the actual Day 1 and Day 3 images (n = 10 animals in each age group). As mentioned above, for the actual images, COR converged to zero at larger *d* values for Day 3 than for Day 1 (Fig. 3b–e). However, COR of Day 3-fied images converged to zero at almost the same *d* as the Day 1 images for the Smoothed and Large Structure patterns (Fig. 6d, e), and the Combination Pattern (Additional file 8: Fig. S5). Therefore, none of the three patterns of Day 3-fied images accurately represented the differences in COR between actual Day 1 and Day 3 images. This finding suggests that the age-associated changes in cytoplasmic texture cannot be artificially reproduced by manipulating the smoothness of the image or adding irregular large structures.

### Synthetic images with different sizes of granules recapitulated the difference in COR between Day 1 and Day 3 oocytes

**Fig. 7:**
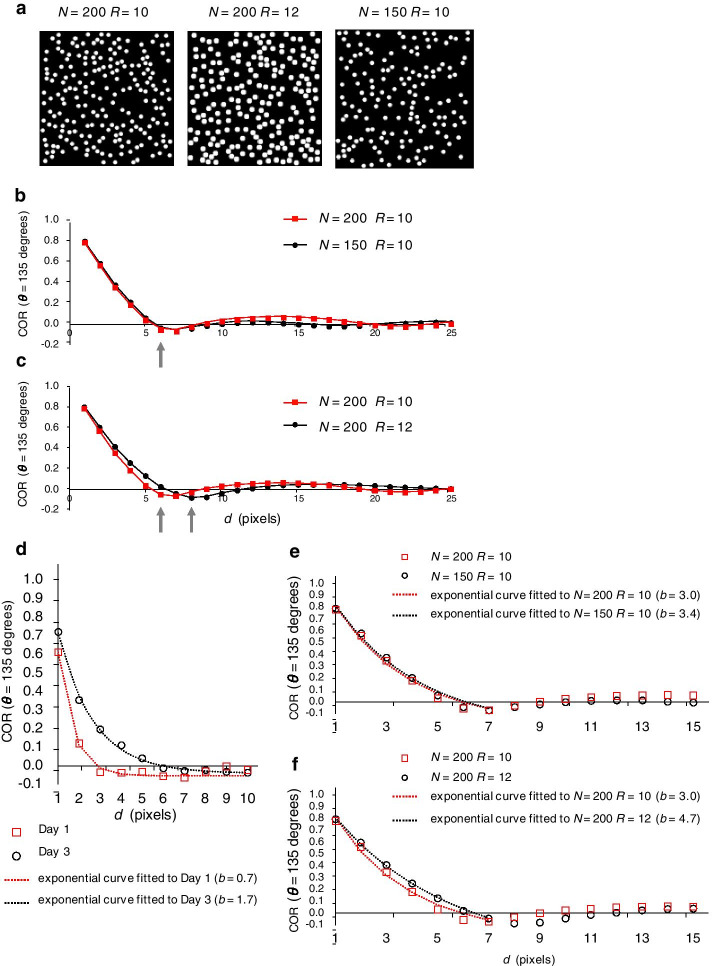
Synthetic images created by using different numbers and sizes of particles. **a** Synthetic images created by setting the number (*N*) and size (*R* pixels) of particles to (left) 200 and 10, (middle) 200 and 12, or (right) 150 and 10, respectively. **b**, **c** Curves of mean COR as a function of distance *d* when *θ* = 135 are shown for synthetic images with different **b** numbers (*N*) or **c** sizes (*R* pixels) of particles. Gray arrows indicate *d* converging to zero. **d** Exponential curves fitted to COR values as a function of *d* when *θ* = 135 are shown for actual Day 1 and Day 3 oocytes (n = 10 animals in each age group; orientation of the worms is 0°). **e**, **f** Exponential curves fitted to COR values as a function of *d* when *θ* = 135 are shown for synthetic images with different **e** numbers (*N*) or **f** sizes (*R* pixels) of particles

To investigate what factor causes the *d* value at which COR converges to zero to differ between Day 1 and Day 3 oocytes, we created simple synthetic images based on two hypotheses: that the (1) number or (2) size of granules in the cytoplasm changes with aging. We therefore evaluated the convergence of COR to zero in the synthetic images with different granule numbers (*N*) or sizes (*R* pixels) (Fig. 7a).

When we varied granule number but kept the granule size constant at *R* = 10, COR in the synthetic images converged to zero at approximately the same *d* regardless of whether 200 or 150 granules were present (Fig. 7b). In contrast, when we varied the granule size but kept the granule number constant at *N* = 200, COR converged at a larger *d* when the granule size was 12 pixels compared with 10 pixels (Fig. 7c). Therefore, merely altering the number of the granules did not recapitulate the difference in the convergence of COR between Day 1 and Day 3 oocytes, but changing the size of cytoplasmic granules did recapitulate this difference.

To objectively assess whether changing granule size in synthetic images yields the anticipated difference in the *d* at which COR converges to zero, we exponentially approximated COR curves by using Eq. (1), where *a* is the amplitude, *b* is a constant that dictates the *d* value at which *f*(*x*) converges to zero, and *c* is the offset.

1 $$f\left(x\right)=a{e}^{-\frac{x}{b}}+c$$

In the approximation function, as *b* increases, the *d* value at which *f*(*x*) converged to zero increases. The difference in the *d* at which COR converges to zero between Day 1 and Day 3 oocytes should reflect the difference in *b* values. When we compared the *b* in the function approximating COR between Day 1 and Day 3 oocytes, the *b* of Day 3 (*b* = 1.7) was larger than that of Day 1 (*b* = 0.7; Fig. 7d). Next, we approximated COR of the synthetic images. The difference in *b* was greater when we manipulated granule size *R* (Fig. 7f; [*N*, *R*] = [200, 10], *b* = 3.0; [*N*, *R*] = [200, 12], *b* = 4.7) than when we varied granule number (*N*) (Fig. 7e; [*N*, *R*] = [200, 10], *b* = 3.0; [*N*, *R*] = [150, 10], *b* = 3.4). The *b* was larger for the larger granule size than the smaller granule size. These results indicate that the difference of the COR property “*d* at which COR converges to zero” between Day 1 and Day 3 oocytes can be reproduced by changing the size of the granules.

### Granules in *C. elegans* oocytes are larger on Day 3 than Day 1

**Fig. 8:**
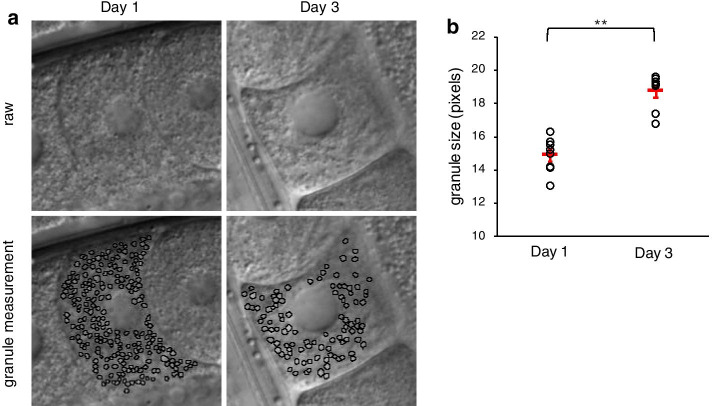
Measurement of the size of cytoplasmic granules in DIC images. **a** Representative DIC images of oocytes. Left, Day 1 oocyte. Right, Day 3 oocyte. Top, Raw images. Bottom, Manual detection of granules on raw images. **b** Comparison of the size of granules manually measured in Day 1 and Day 3 oocytes. The sizes of many of the elongated and clustered granules in Day 3 oocytes were not measured. Circles indicate individual animals (n = 8 animals in each age group); red bars indicate the mean value. Error bars indicate SEM. Asterisks indicate statistical significance (**P < 0.01; Welch’s two-tailed *t* test)

In the synthetic images, changing the granule size reproduced the difference in COR between Day 1 and Day 3 oocytes, suggesting that cytoplasmic granules in *C. elegans* oocytes might change in size with aging. To examine whether granules in DIC images demonstrated age-associated size variation, we manually measured granules and compared their size on Days 1 and 3 (n = 8 animals in each age group; Fig. 8a). Granules were significantly larger in Day 3 oocytes than Day 1 oocytes (Fig. 8b).

## Discussion

In this study, we found that the texture of the cytoplasm in *C. elegans* oocytes varies with their age. These changes were characterized quantitatively through the DIC image features of Mm Value and COR, the second of which is based on the second-order statistic GLCM. In addition, with the use of appropriate parameter sets, COR characterized these age-associated changes in texture more significantly than Mm Value. Furthermore, analysis of synthetic images and measurement of the size of cytoplasmic granules suggested that the cytoplasmic granules in *C. elegans* oocytes become larger with aging.

Mm Value, a measure of texture roughness, is calculated as the mean of the difference between the maximum and minimum intensities within successive moving windows. The statistical significance of the difference in Mm Values between Day 1 and Day 3 oocytes decreased as the window size increased (Fig. 2e). If the texture contrast is uniform within an oocyte, calculating Mm Value by using a window size that is smaller than the granule size enables the Mm Value to fluctuate depending on the size or density of granules. However, this variation in the Mm Value cannot occur when the window size used for determining Mm Value might contains multiple granules, such as 13 × 13, 15 × 15, or 17 × 17 pixels. Mm Value for Day 1 oocytes remained significantly larger than that for Day 3 oocytes for these window sizes (*P* values < 0.05; Fig. 2e). Therefore, texture contrast might decrease with aging. However, the utility of Mm Value for characterizing the age-associated changes in cytoplasmic texture disappeared when window size was set to 19 × 19 pixels or larger (all *P* values > 0.05; Fig. 2e). When determined by using windows sufficiently large to contain multiple granules, the *P* values for age-associated differences in Mm Value increased as the window size increased. This may indicate that texture contrast changes in a spatially inhomogeneous manner.

Regardless of window size, the Mm Values of the Day 3-fied images based on the Smoothed pattern were significantly smaller than those of Day 1 images (Additional file 9: Fig. S6a). In addition, the Mm Values of the Day 3 images did not differ significantly from those of Day 1 images when the window was 25 × 25 pixels or larger. Furthermore, contrast in the Smoothed pattern image was decreased due to normalization to the overall texture contrast throughout the image. Therefore, the results suggest that texture contrast in oocytes does not change homogeneously from Day 1 to Day 3. The relationship between the Mm Values of Day 1 and 3 images may reflect the inhomogeneous changes of the texture contrast. Given that changes in the optical phase gradient can alter contrast in DIC images, the age-associated decrease in texture contrast might reflect a change in granule content due to chemical modification or a difference in content quantity. Similar to the results for Mm Values of Day 3 images, the Mm Values of Day 3-fied images with the Large Structure and Combination patterns were not significantly different from Day 1 images when the window was 25 × 25 pixels or larger (Additional file 9: Fig. S6b and c). The Day 3-fied images based on the Large Structure and Combination patterns were created by changing the contrast of the Day 1 image in a spatially inhomogeneous manner.

Compared with SD and entropy, the Mm value characterized the age-associated differences in cytoplasmic texture between Day 1 and 3 oocytes for a broader range of window size (Fig. 2e–g; Additional file 5: Fig. S2). SD and entropy were calculated by using the grey values of all pixels in the moving window, whereas the Mm Value was calculated by using the grey values of only the maximum and minimum intensities in the moving window. The applicability of the Mm Value across many window sizes may stem from the use of these two extreme values.

COR significantly characterized age-associated variations between the Day 1 and Day 3 oocytes when using smaller *d* but not or less significantly when using larger *d* (Fig. 3b–e). This finding suggests some small changes in structure with aging. Therefore, COR likely characterized age-associated changes in texture by recognizing small structures in the cytoplasm.

The *d* value at which COR converged to zero was larger in Day 3 oocytes than in Day 1 oocytes (Fig. 3b–e). The *d* at which COR converged to zero in Day 3-fied images differed markedly from that in actual Day 3 images (Fig. 6d and e), and the *d* at which COR converged to zero in the synthetic images was affected by the size of the granules rather than their quantity (Fig. 7b and c). These results suggest that granule size is the major factor affecting the convergence of COR to zero, and that the difference in the convergence properties of COR between Day 1 and Day 3 images may reflect the larger size of granules in Day 3 oocytes. Supporting this notion, we found that cytoplasmic granules in Day 3 oocytes were significantly larger than those in Day 1 oocytes (Fig. 8b); i.e., cytoplasmic granules in *C. elegans* oocytes become larger with age.

At the optimal parameter setting (*d* = 1, *θ* = 135) and a window of 3 × 3 pixels, COR characterized cytoplasmic texture with a lower *P* value than that obtained with the Mm Value. However, COR was not always superior to Mm value because the effectiveness of COR depended on the angle *θ* and distance *d*. The results of image analysis of rotated worms suggest that the age-associated differences in texture include an angle-dependent property that is intrinsic to the DIC imaging system. The results of analyzing rescaled images suggest that the age-associated differences also include a size-dependent property intrinsic to the resolution of the imaging system. At appropriate parameters, COR characterized the changes in texture with lower *P* values than those obtained with Mm Value, but Mm Value was more informative at a broader range of parameters and was not particularly influenced by the angle-dependent property of DIC images. Mm Value may detect a feature of the age-associated changes that COR cannot detect. Further studies are needed to clarify the difference in the characteristics of the age-associated changes detected by Mm Value and COR.

Reported age-associated changes in the morphologic appearance of *C. elegans* oocytes include oocyte shrinkage, loosened contacts, and aggregation into large clusters [5, 9]. Here we have quantitatively characterized age-associated changes in the cytoplasmic texture of *C. elegans* oocytes through several statistical image features, such as Mm Value and COR. Cytoplasmic texture would reflect the internal status of oocytes more directly than the external morphologic appearance. Quantitative analysis of cytoplasmic texture and measurement of granules suggest that the cytoplasmic granules in *C. elegans* oocytes become larger with aging. Mm Value and COR can be used as objective methods to quantify age-associated differences in oocyte appearance, which may reflect oocyte fertility. To use the image features to classify the texture of oocytes according to their age, image features should differ markedly relative to oocyte age. Although Mm value and COR characterized age-associated changes in cytoplasmic texture, their distribution overlapped between age groups, and the values differed significantly between Day 1 and Day 3 oocytes but not between Day 1 and Day 2 oocytes. These results suggest that Mm value and COR may be insufficiently sensitive for accurate recognition and classification of small textural changes. To increase sensitivity, multi-dimensional analysis using additional image features or application of machine learning methods, such as deep learning, may be required. The age-associated changes in cytoplasmic texture can be subtle.

COR was similar on Day 1 and Day 2, and significantly increased on Day 3. Mm value was similar on Day 1 and Day 2, and significantly decreased on Day 3. A possible explanation of these results is that the age-related change in oocyte quality is not reflected in the cytoplasmic texture of oocytes between Day 1 and Day 2. Alternatively, there could be almost no age-related change in the oocyte quality between Day 1 and Day 2. Given that almost all of the fertilized embryos developed successfully in the Day 1 and Day 2 adults (Additional file 1: Movie S1 and Additional file 2: Movie S2), but not the Day 3 adults (Additional file 3: Movie S3), it is likely that oocyte quality does not change much between Day 1 and Day 2, but remarkably decreases on Day 3.

Some of the cytoplasmic granules in our DIC texture images may be yolk granules [26, 27]. Yolk is a lipoprotein composed of lipids and lipid-binding proteins called vitellogenins [28]. In *C. elegans*, vitellogenins are synthesized in the intestine and transported into maturing oocytes through endocytosis [29, 30]. Yolk provides essential nutrients to the eggs to support embryonic development [30]. During reproductive senescence, the intestine continues to produce and secrete large amounts of yolk protein. In adult *C. elegans* hermaphrodites, yolk accumulates towards the end of the self-fertile reproductive period [31, 32]. Provisioning of vitellogenin to embryos increases with maternal age [33] and might increase the lipid content in embryos and oocytes, given that vitellogenins transport lipids into embryos [28, 33]. Taking these findings together with our data, we propose that the cytoplasmic granules in aged adults (Day 3 and later) might enlarge due to an increase in vitellogenin content or in vitellogenin-transported embryonic lipid content.

What is the relationship between decreased fertility and yolk accumulation with aging? Yolk accumulation may contribute to the decrease in fertility. High levels of yolk appear to be detrimental and decrease the lifespan of *C. elegans* [34]. In contrast, knockdown of vitellogenin expression extends lifespan [35, 36]. Increased amounts of yolk might accelerate the aging of oocytes or animals, resulting in decreased fertility. Alternatively, decreased oocyte fertility might contribute to yolk accumulation. Moreover, we cannot exclude the possibility that yolk accumulation does not affect fertility directly. Further experiments are needed to clarify the relationship between yolk levels and fertility in this and other species.

## Conclusions

Here, we found that the Mm Value and COR objectively quantify age-related changes in *C. elegans* oocyte in Nomarski DIC microscopy images. We are planning to apply these image features to publicly available Nomarski DIC microscopy images of *C. elegans* oocytes and embryos in public image databases such as Phenobank [37] and WDDD [38]. Such applications may provide clues to the molecular mechanisms of oocyte aging.

## Methods

### *Caenorhabditis elegans* strains and growth conditions

*Caenorhabditis elegans* (Bristol N2 strain) were grown under standard conditions [39]. The L4 larval stage was considered as Day 0; worms were defined as Day 1, Day 2, and Day 3 adults at 18–24 h, 43–46 h, and 67–70 h after L4, respectively.

### Imaging of oocytes

Worms were immobilized in a polystyrene nanoparticle suspension [40] supplemented with 5-hydroxytryptamine [41] on agarose pads. The anterior gonad was observed by Nomarski DIC microscopy with the use of a Leica HCX PL APO 63 × /1.20 W CORR objective and an iXonX3 electron-multiplying CCD camera controlled with live-cell imaging software (Andor iQ). The plane of focus was through the oocyte nucleus. Images of nematodes at four angles (0, 45, 90, and 135°) were obtained by rotating the Day 1 samples; 0° was defined as horizontal orientation. Digital images of 512 × 512 pixels were converted to 14-bit TIFF format (0.25 μm per pixel).

### Calculation of image features

To calculate image features, random regions of 30 × 30 pixels were extracted from the cytoplasm, without including nuclei or cell boundaries. The 3 oocytes most proximal to the spermatheca in the anterior gonad were used for each animal, and one region was extracted from each oocyte. Image features of individual animals were defined as the mean of those in the extracted three regions.

The first-order statistical features Mm Value, SD, and entropy were calculated by moving the local window within the confines of the border of the extracted region. SD was defined as the mean of the standard deviations of the pixel intensities in the moving window. Entropy was defined as the mean of entropies (calculated according to the following the equation) in the moving window:

$$-\sum_{k=0}^{\mathrm{G}-1}P\left(k\right){\mathrm{log}}_{2}P\left(k\right)\text{,}$$

where G is the number of gray levels, and *P*(*k*) is the probability of occurrence of gray level *k* in the moving window.

Second-order statistical features based on GLCM—*Correlation* (COR), *Angular Second Moment* (ASM), *Contrast* (CON), *Inverse Difference Moment* (IDM), and *Entropy* (ENT)—were calculated by using the following equations and the co-occurrence matrix *P*(*i, j* | *d, θ*):

$${\text{COR}}= \frac{{\sum }_{i=0}^{\mathrm{G}-1}{\sum }_{j=0}^{\mathrm{G}-1}ijP\left(i,j\right) - {\mu }_{x}{\mu }_{y}}{{\sigma }_{x}{\sigma }_{y}}$$

$$\text{ASM }\text{=} {\sum }_{i=0}^{\mathrm{G}-1}{\sum }_{j=0}^{\mathrm{G}-1}{\left\{P\left(i,j\right)\right\}}^{2}$$

$${\text{CON}} = {\sum }_{i=0}^{\mathrm{G}-1}{\sum }_{j=0}^{\mathrm{G}-1}{\left(i-j\right)}^{2}P\left(i,j\right)$$

$${\text{IDM}} = {\sum }_{i=0}^{\mathrm{G}-1}{\sum }_{j=0}^{\mathrm{G}-1}\frac{P\left(i,j\right)}{1+{\left(i-j\right)}^{2}}\text{, and}$$

$${\text{ENT}}= -{\sum }_{i=0}^{\mathrm{G}-1}{\sum }_{j=0}^{\mathrm{G}-1}P\left(i,j\right)\mathrm{log}\left(P\left(i,j\right)\right)\text{,}$$

where ${\mu }_{x}$, ${\mu }_{y}$, ${\sigma }_{x}$, and ${\sigma }_{y}$ are the means and standard deviations in the *x* and *y* direction given by

$${\mu }_{x}= {\sum }_{i=0}^{\mathrm{G}-1}{\sum }_{j=0}^{\mathrm{G}-1}iP\left(i,j\right)\text{,}{ \mu }_{y}= {\sum }_{i=0}^{\mathrm{G}-1}{\sum }_{j=0}^{\mathrm{G}-1}jP\left(i,j\right) {\text{and}}$$

$${{\sigma }_{x}}^{2}= {\sum }_{i=0}^{\mathrm{G}-1}{\sum }_{j=0}^{\mathrm{G}-1}{P\left(i.j\right)}{\left(i-{\mu }_{x}\right)}^{2}\text{,} { {\sigma }_{y}}^{2}= {\sum }_{i=0}^{\mathrm{G}-1}{\sum }_{j=0}^{\mathrm{G}-1}{P\left(i.j\right)}{\left(j-{\mu }_{y}\right)}^{2}\text{.}$$

*P*(*i, j* | *d, θ*) was defined as symmetric (see Fig. 3a).

### Computational complexity

The computational complexity of the Mm Value is of the order of *O*(W^2^M^2^), where W is the window size, and M is the size of the input image. The computational complexity of the COR is divided into two components: (1) creating the GLCM and (2) calculating the COR from the GLCM [42]; the computational complexity is of the order of *O*(L^2^) + *O*(G^2^), where L is the length of the neighborhood window in GLCM feature extraction, and G is the number of grey levels of the input image. Using our codes, it takes about 0.5 × 10^–3^ s per image to calculate the Mm Value for W = 3 pixels and M = 30 pixels on our PC (Intel® Core™ i5, 1.6 GHz). The calculation of the COR for L = 1 pixel and G = 256 takes about 2.4 × 10^–3^ s per image on our PC (Additional file 10: Table S1).

### Creation of the rescaled images

The images were rescaled using bilinear interpolation. We used data from worms oriented at 0° (n = 10 animals) as the original images (30 × 30 pixels). To halve the resolution of the original images, the images were downscaled to 15 × 15 pixels. To double the resolution of the original images, the images were upscaled to 60 × 60 pixels.

### Creation of the synthetic images

Synthetic images were created by randomly locating *N* white granules with diameter *R* pixels on a black background image of 300 × 300 pixels. Granules could not overlap or protrude from the border of the image.

### Fitting equations to the COR curves

To fit equations to the COR datasets (actual Day 1 and Day 3 images, and synthetic images) we performed nonlinear regression analyses using the exponential function described in the Results section:

$$f\left(x\right)=a{e}^{-\frac{x}{b}}+c$$

We used the COR data for *d* of 1–7 pixels to estimate the curve for convergence of COR to zero. We used data from worms oriented to 0° (n = 10 animals).

### Quantification of the granule size

For each worm, we manually measured the cytoplasmic granules in the oocyte that was second-most proximal to the spermatheca. Granules were determined as non-overlapping circular or nearly circular regions where signal intensity exceeded the background intensity. Many of the elongated and clustered granules in Day 3 oocytes were not measured.

### Statistical analyses

Statistical analyses were performed using R software. *P* values for pair-wise comparisons of data sets were calculated using Welch’s two-tailed *t* test. Those for multiple comparisons of data sets were calculated using Tukey–Kramer test.

## Supplementary Information

**Additional file 1: Movie S1**. Fertilization in 1-day-old *C. elegans* adults observed by using Nomarski DIC microscopy. A worm was filmed at the start of fertilization. The time-lapse image was captured at a frame interval of 60 s using a Leica DM6000B microscope with HCX PL FLUOTAR 40×/0.75 objective at 0.16 μm per pixel.

**Additional file 2: Movie S2**. Fertilization in 2-day-old *C. elegans* adults observed by using Nomarski DIC microscopy. A worm was filmed about 24 h after the start of fertilization. The time-lapse image was captured under the same conditions as used for the Day 1 adults (Additional file 1: Movie S1).

**Additional file 3: Movie S3**. Fertilization in 3-day-old *C. elegans* adults observed by using Nomarski DIC microscopy. A worm was filmed about 48 h after the start of fertilization. The time-lapse image was captured under the same conditions as used for Day 1 adults (Additional file 1: Movie S1).

**Additional file 4: Figure S1**. Comparison of the Max–min Value (Mm Value) between Day 1, Day 2, and Day 3 oocytes in each of two experiments. Mm Value was calculated by using a 3 × 3-pixel window in Day 1, Day 2, and Day 3 oocytes. Circles indicate individual animals (n = 4 or 8 animals each age group); red bars indicate the mean values. Error bars indicate SEM. Asterisks indicate statistical significance (*P < 0.05; Tukey–Kramer test).

**Additional file 5: Figure S2**. Comparison of **a** Mm Value, **b** SD, and **c** entropy between Day 1 and Day 3 oocytes. Various window sizes from 3 × 3 to 29 × 29 pixels were used. Data are means ± SEM (n = 10 animals in each age group). Asterisks indicate statistical significance between Day 1 and Day 3 oocytes (*P < 0.05; **P < 0.01; Welch’s two-tailed *t* test).

**Additional file 6: Figure S3**. Comparison of key texture features based on a Gray Level Co-occurrence Matrix (GLCM) between Day 1, Day 2, and Day 3 oocytes. Curves of the indicated texture features as a function of distance *d* when *θ* = 135 are shown. Data are means ± SEM (n = 12 animals each age group, pooled from two experiments). ASM, Angular Second Moment; CON, Contrast; IDM, Inverse Difference Moment; ENT, Entropy; COR, Correlation. Symbols indicate statistical significance (Tukey–Kramer test) between Day 1 and Day 3 oocytes (*P < 0.05; **P < 0.01) or Day 2 and Day 3 oocytes (^†^*P* < 0.05, ^††^*P* < 0.01).

**Additional file 7: Figure S4**. Comparison of SD and entropy between actual images and Day 3-fied images. **a** SD and **b** entropy of actual Day 1, actual Day 3, and the three patterns of Day 3-fied images were calculated using a window size of 3 × 3-pixels. The Smoothed pattern parameter *offset* was set to 50, 100, and 200. The Large Structure pattern parameter *iteration* was set to 1, 3, and 5. The Combination pattern parameters (*offset*, *iteration*) were set to (50, 1), (50, 3), and (50, 5). Circles indicate the image features of individual animals (n = 10 animals in each age group; orientation of the worms is 0°); red bars indicate the mean values. Error bars indicate SEM. Symbols indicate statistical significance (Tukey–Kramer test) between actual Day1 and actual Day 3 or Day 3-fied images (**P < 0.01) or between actual Day 3 and Day 3-fied images (^†^*P* < 0.05; ^††^*P* < 0.01); N.S., no significant difference between actual Day 3 and Day 3-fied images.

**Additional file 8: Figure S5**. *Correlation* (COR) calculated for Day 1 and Day 3 oocyte images and Day 3-fied oocyte images with two different Combination patterns. Curves of mean COR as a function of distance *d* when *θ* = 135 are shown. Data are means ± SEM (n = 10 animals each age group; orientation of the worms is 0°). The parameters for the Combination pattern (*offset* and *iteration*) were set to **a** (50, 5) and (200, 5) or **b** (200, 1) and (200, 5), respectively.

**Additional file 9: Figure S6**. Comparison of Mm Values between actual Day 1, actual Day 3, and Day 3-fied oocyte images for various window sizes from 3 × 3 to 29 × 29 pixels. Data are means ± SEM (n = 10 animals each age group; orientation of the worms is 0°) **a** Day3-fied images were created with Smoothed pattern (*offset* was set to 100).**b** Day3-fied images were created with Large Structure pattern (*iteration* was set to 3). **c** Day3-fied images were created with Combination pattern [(*offset*, *iteration*) were set to (50, 1)]. Symbols indicate statistical significance (Tukey–Kramer test) between Day 1 and Day 3 images (*P < 0.05; **P < 0.01) or Day 1 and Day 3-fied images (^†^*P* < 0.05; ^††^*P* < 0.01).

**Additional file 10: Table S1**. Processing time per image for Mm Value, SD, entropy and COR. The processing time was measured for input images of various sizes. The parameter settings were changed according to the image size. Processing time per image was calculated by averaging the processing time of 1000 images. The processing time for Mm Value was equivalent to that for SD, and that for entropy was greater than that for Mm Value or SD. The computational complexities of the SD and entropy are of the order of *O*(W2M2) and *O*(W2M2) + *O*(GM2), respectively, where W is the window size, M is the size of the input image and G is the number of grey levels of the input image. The processing time for the first-order statistical features was less than that for COR when the input images were small (30 × 30 pixels), but greater than that for COR when the input images were large (150 × 150 pixels). This was because the processing times for the first-order statistical features increased with the image size, but the processing time for COR was largely unaffected by the image size.

## Data Availability

The oocyte images are available at the Systems Science of Biological Dynamics database (SSBD) [43], 10.24631/ssbd.repos.2020.07.001.

## Abbreviations

COR: Correlation
DIC microscopy: Differential interference contrast microscopy
GLCM: Gray level co-occurrence matrix
Mm Value: Max–min value
