# Supplementary figures and images for: Statistical image processing quantifies the changes in cytoplasmic texture associated with aging in Caenorhabditis elegans oocytes
Source: BMC Bioinformatics. 2021 Feb 17;22:73. doi: 10.1186/s12859-021-03990-3 (PMC7890843; doi:10.1186/s12859-021-03990-3)

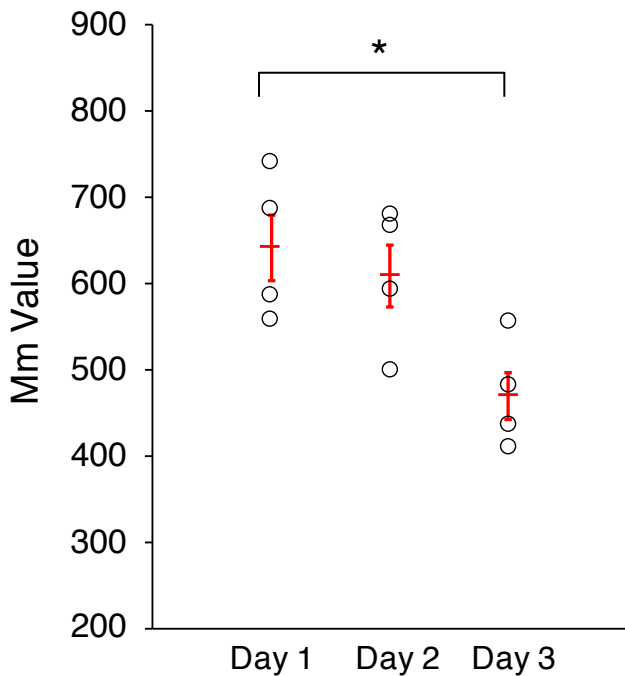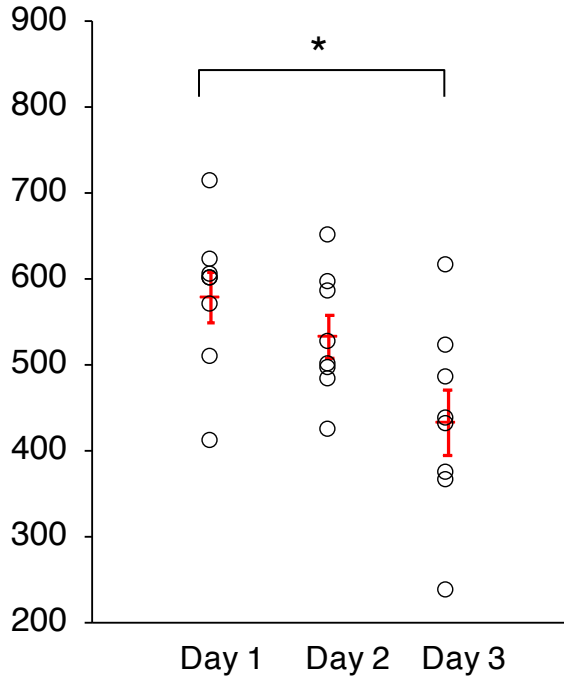

Supplement: Supplementary file 4 — Additional file 4: Figure S1. Comparison of the Max–min Value (Mm Value) between Day 1, Day 2, and Day 3 oocytes in each of two experiments. Mm Value was calculated by using a 3 × 3-pixel window in Day 1, Day 2, and Day 3 oocytes. Circles indicate individual animals (n = 4 or 8 animals each age group); red bars indicate the mean values. Error bars indicate SEM. Asterisks indicate statistical significance (*P < 0.05; Tukey–Kramer test). [file 12859_2021_3990_MOESM4_ESM.pdf]

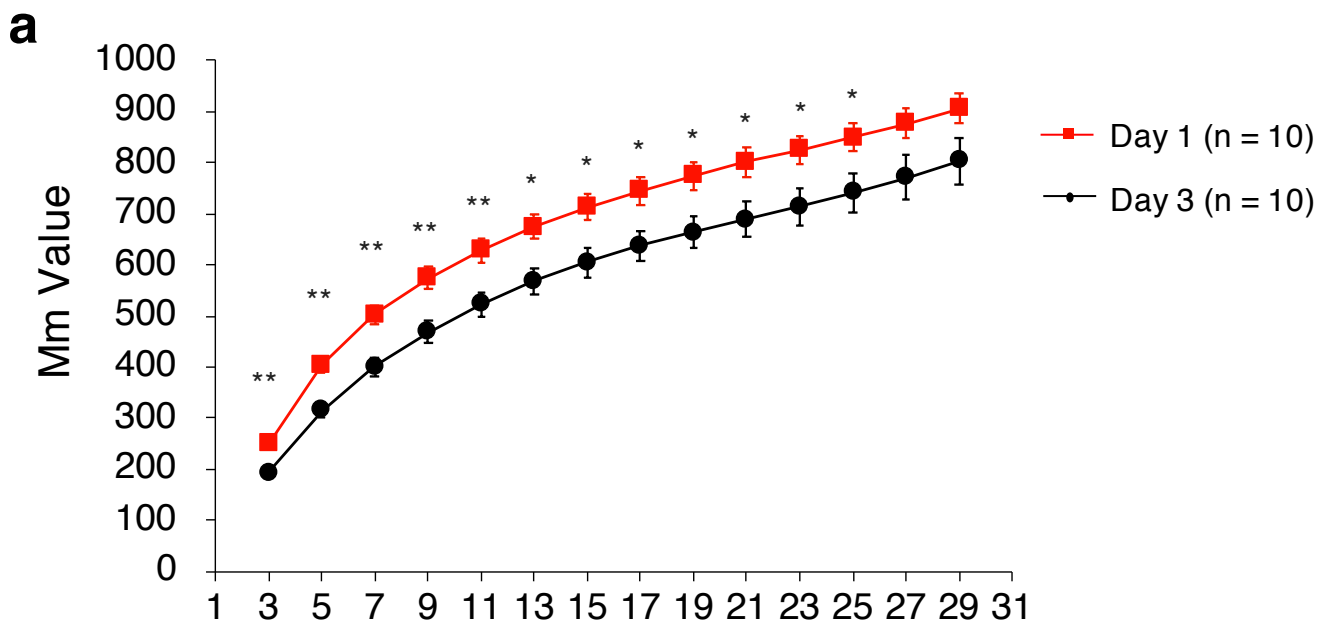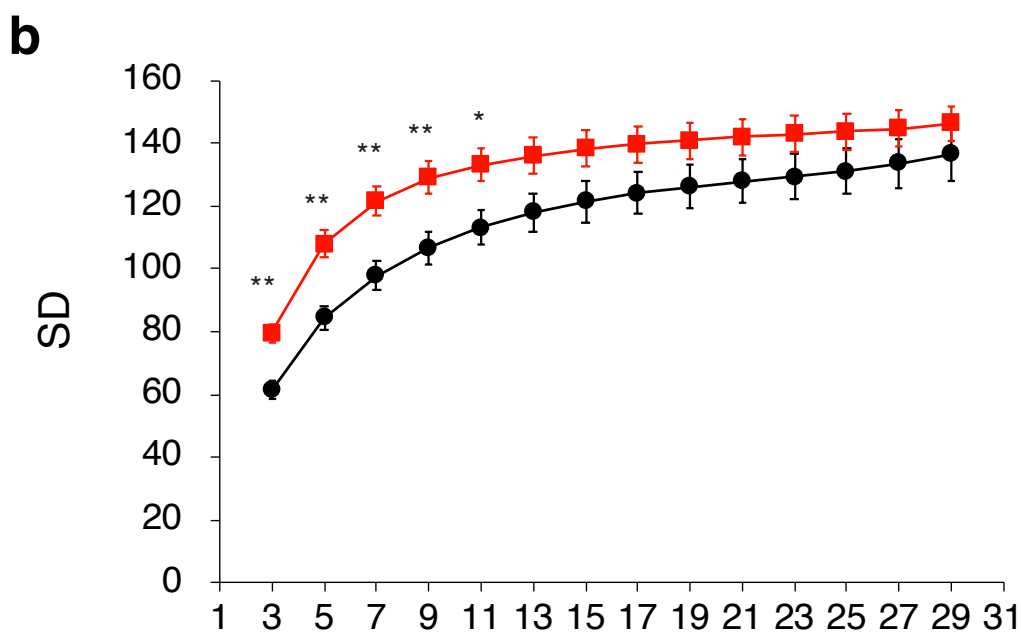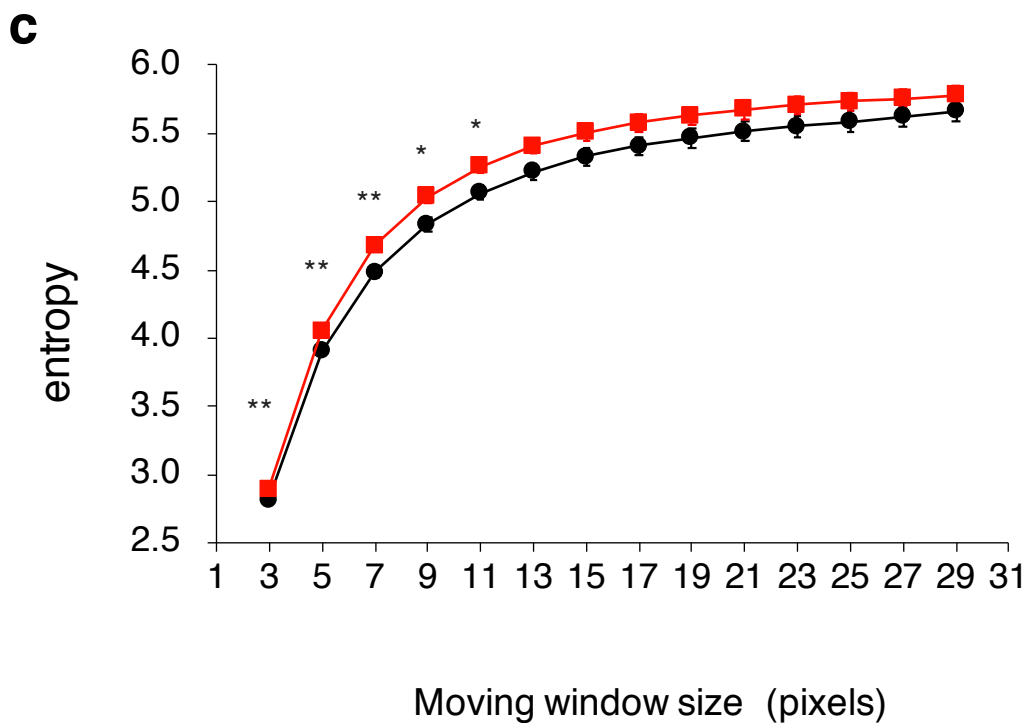

Supplement: Supplementary file 5 — Additional file 5: Figure S2. Comparison of a Mm Value, b SD, and c entropy between Day 1 and Day 3 oocytes. Various window sizes from 3 × 3 to 29 × 29 pixels were used. Data are means ± SEM (n = 10 animals in each age group). Asterisks indicate statistical significance between Day 1 and Day 3 oocytes (*P < 0.05; **P < 0.01; Welch’s two-tailed t test). [file 12859_2021_3990_MOESM5_ESM.pdf]

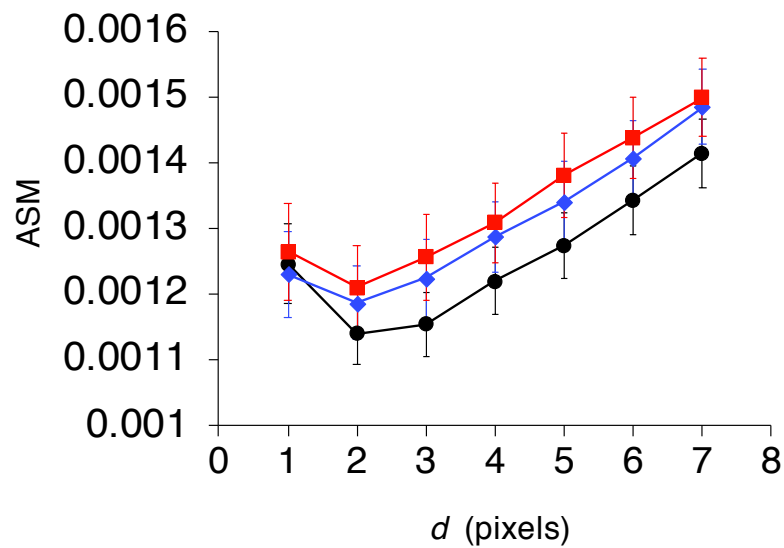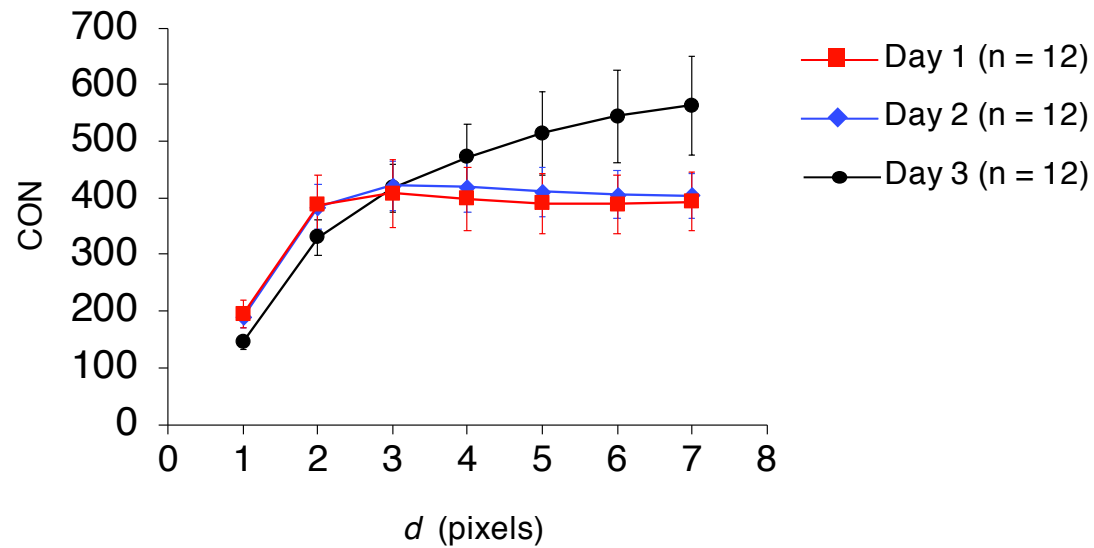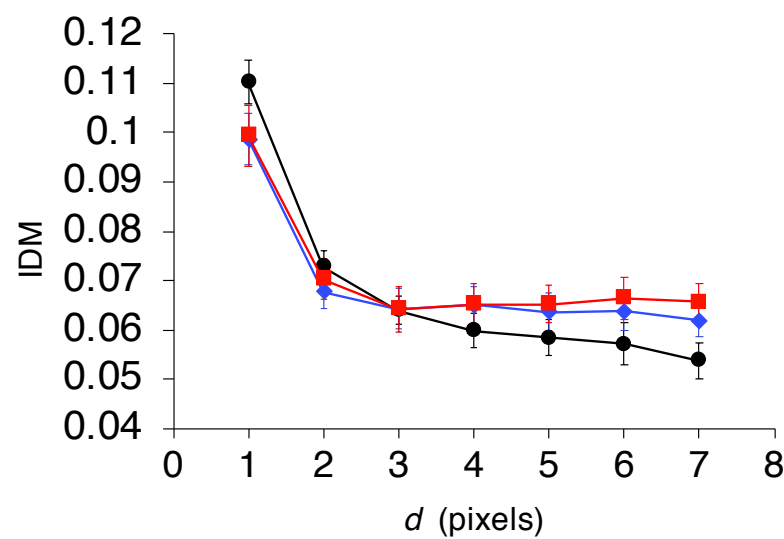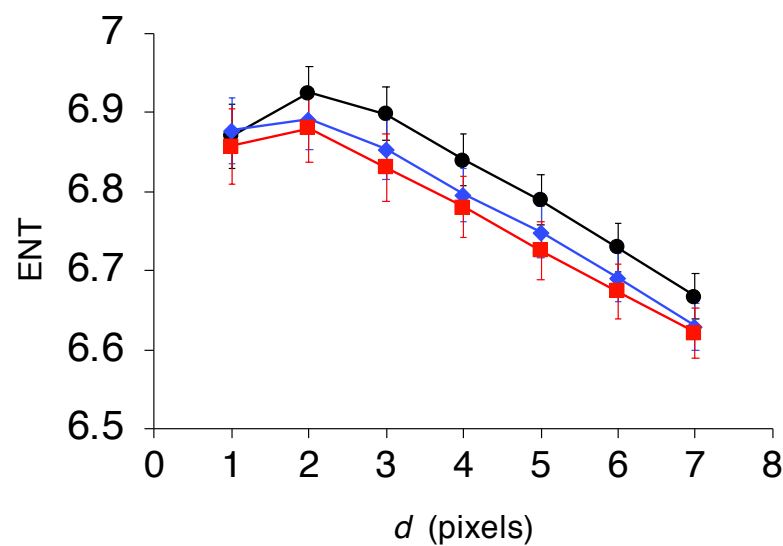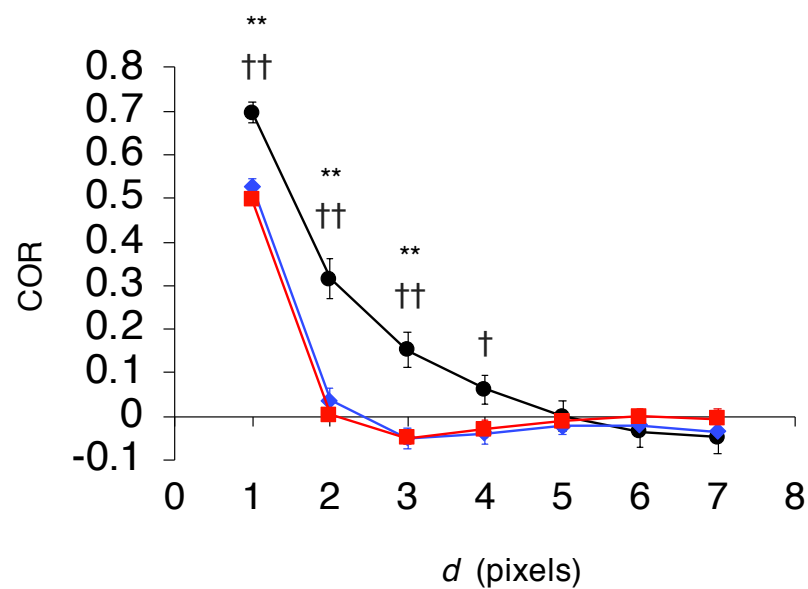

Supplement: Supplementary file 6 — Additional file 6: Figure S3. Comparison of key texture features based on a Gray Level Co-occurrence Matrix (GLCM) between Day 1, Day 2, and Day 3 oocytes. Curves of the indicated texture features as a function of distance d when θ = 135 are shown. Data are means ± SEM (n = 12 animals each age group, pooled from two experiments). ASM, Angular Second Moment; CON, Contrast; IDM, Inverse Difference Moment; ENT, Entropy; COR, Correlation. Symbols indicate statistical significance (Tukey–Kramer test) between Day 1 and Day 3 oocytes (*P < 0.05; **P < 0.01) or Day 2 and Day 3 oocytes (†P < 0.05, ††P < 0.01). [file 12859_2021_3990_MOESM6_ESM.pdf]

**a**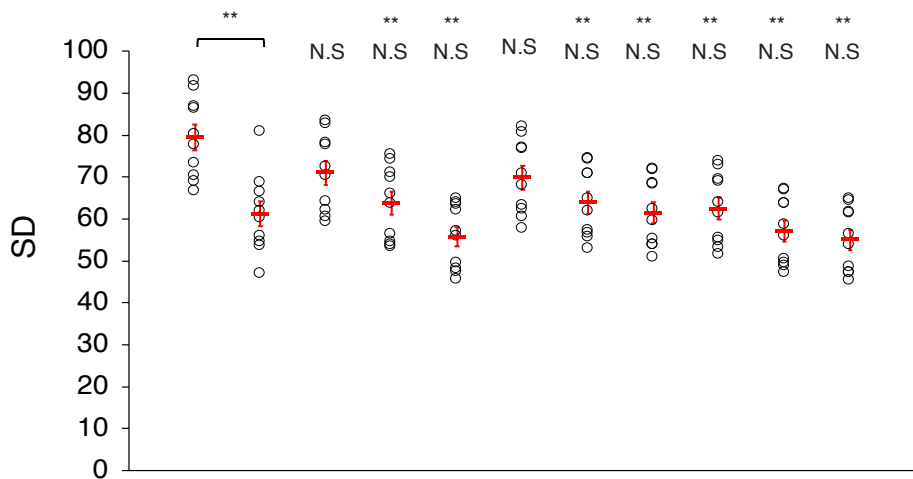**b**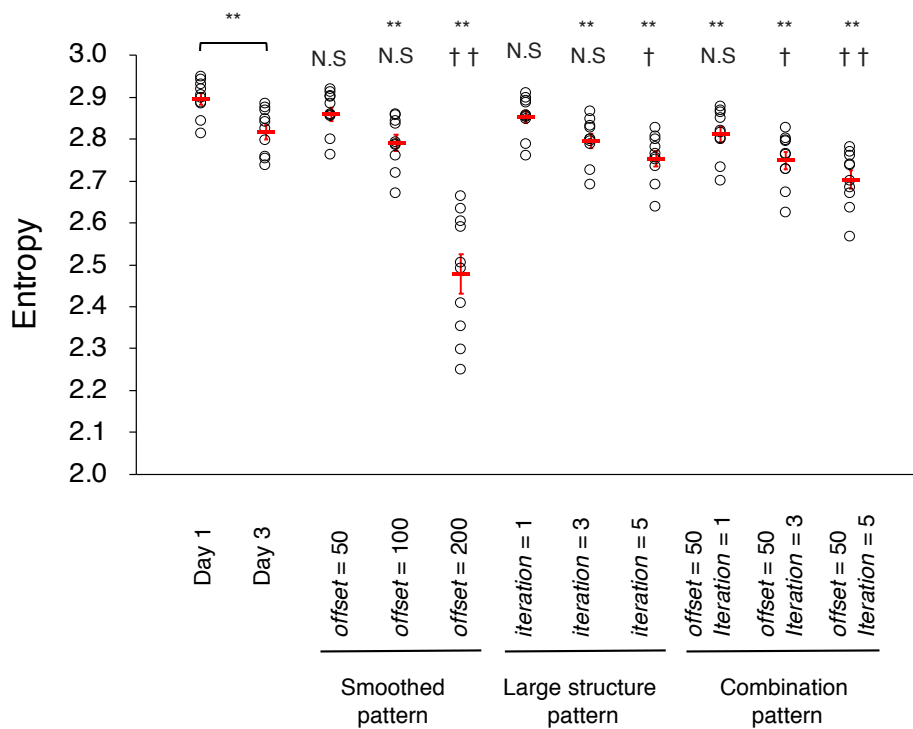

Supplement: Supplementary file 7 — Additional file 7: Figure S4. Comparison of SD and entropy between actual images and Day 3-fied images. a SD and b entropy of actual Day 1, actual Day 3, and the three patterns of Day 3-fied images were calculated using a window size of 3 × 3-pixels. The Smoothed pattern parameter offset was set to 50, 100, and 200. The Large Structure pattern parameter iteration was set to 1, 3, and 5. The Combination pattern parameters (offset, iteration) were set to (50, 1), (50, 3), and (50, 5). Circles indicate the image features of individual animals (n = 10 animals in each age group; orientation of the worms is 0°); red bars indicate the mean values. Error bars indicate SEM. Symbols indicate statistical significance (Tukey–Kramer test) between actual Day1 and actual Day 3 or Day 3-fied images (**P < 0.01) or between actual Day 3 and Day 3-fied images (†P < 0.05; ††P < 0.01); N.S., no significant difference between actual Day 3 and Day 3-fied images. [file 12859_2021_3990_MOESM7_ESM.pdf]

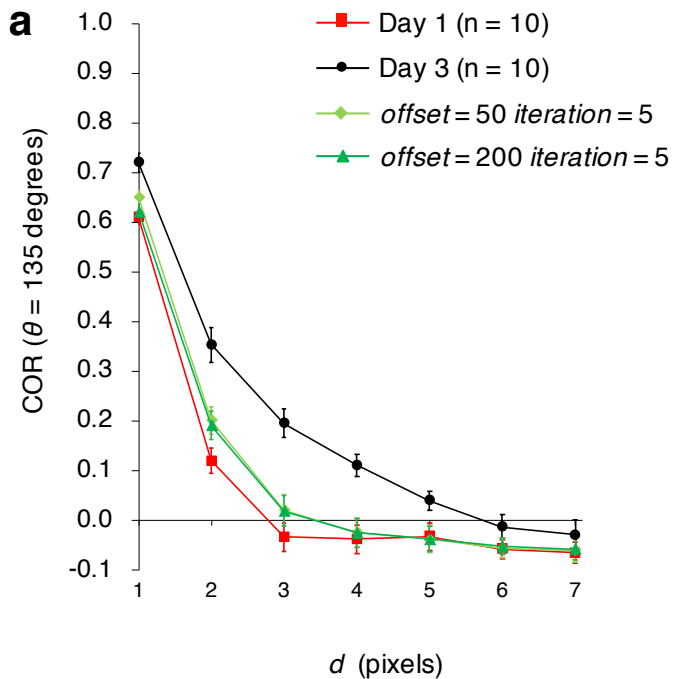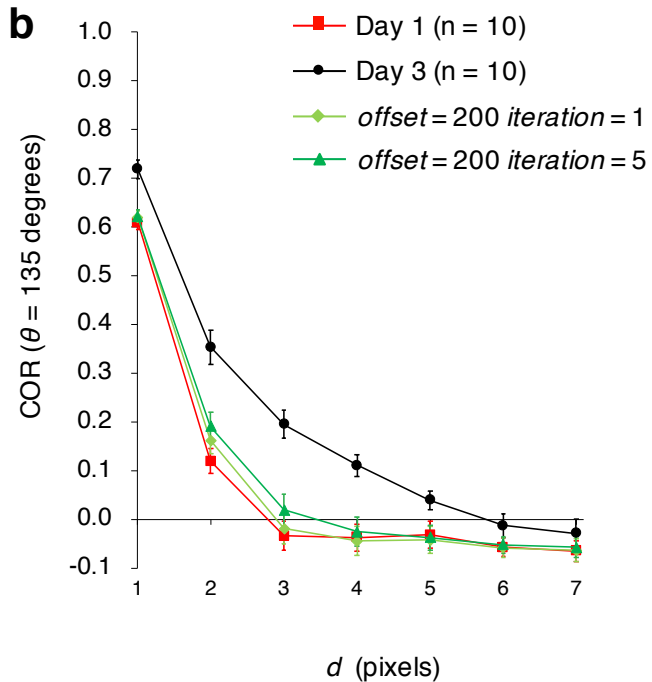

Supplement: Supplementary file 8 — Additional file 8: Figure S5. Correlation (COR) calculated for Day 1 and Day 3 oocyte images and Day 3-fied oocyte images with two different Combination patterns. Curves of mean COR as a function of distance d when θ = 135 are shown. Data are means ± SEM (n = 10 animals each age group; orientation of the worms is 0°). The parameters for the Combination pattern (offset and iteration) were set to a (50, 5) and (200, 5) or b (200, 1) and (200, 5), respectively. [file 12859_2021_3990_MOESM8_ESM.pdf]

**a**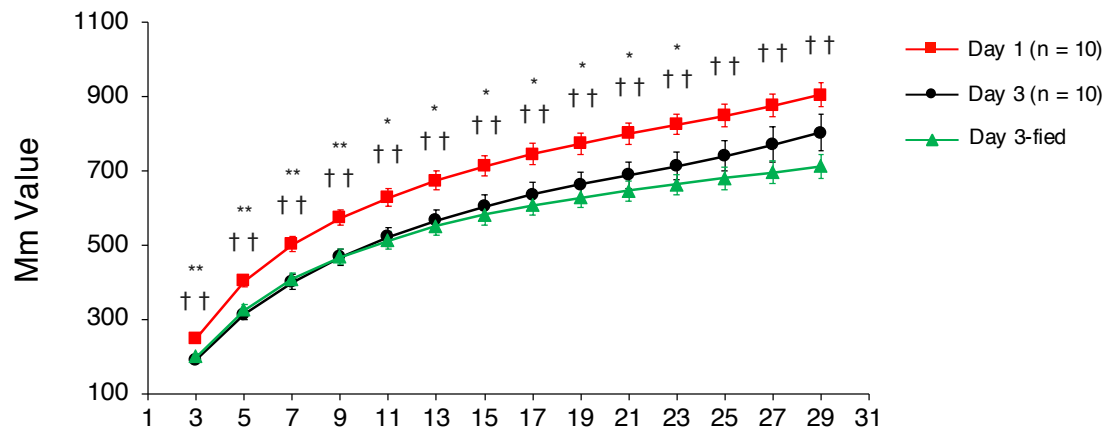**b**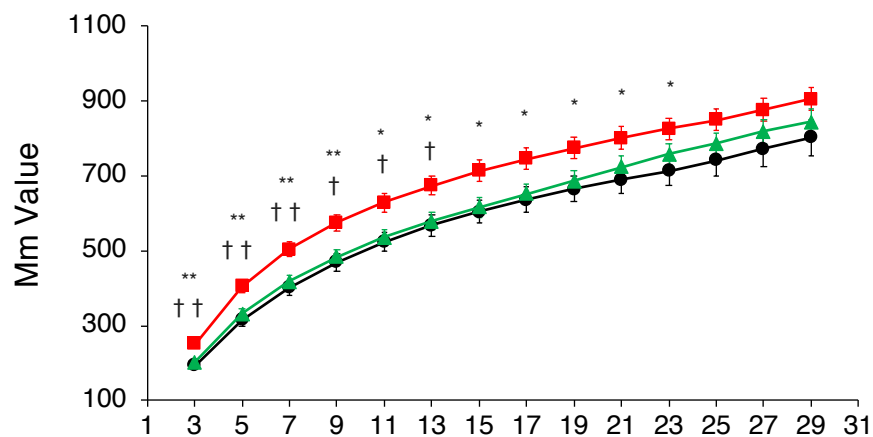**c**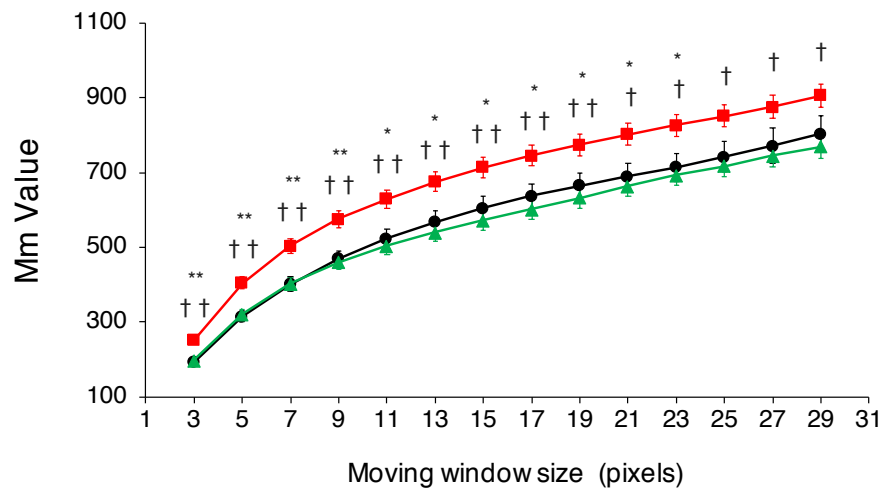

Supplement: Supplementary file 9 — Additional file 9: Figure S6. Comparison of Mm Values between actual Day 1, actual Day 3, and Day 3-fied oocyte images for various window sizes from 3 × 3 to 29 × 29 pixels. Data are means ± SEM (n = 10 animals each age group; orientation of the worms is 0°) a Day3-fied images were created with Smoothed pattern (offset was set to 100).b Day3-fied images were created with Large Structure pattern (iteration was set to 3). c Day3-fied images were created with Combination pattern [(offset, iteration) were set to (50, 1)]. Symbols indicate statistical significance (Tukey–Kramer test) between Day 1 and Day 3 images (*P < 0.05; **P < 0.01) or Day 1 and Day 3-fied images (†P < 0.05; ††P < 0.01). [file 12859_2021_3990_MOESM9_ESM.pdf]
